# Exceptional response and multisystem autoimmune-like toxicities associated with the same T cell clone in a patient with uveal melanoma treated with immune checkpoint inhibitors

**DOI:** 10.1186/s40425-019-0533-0

**Published:** 2019-03-04

**Authors:** Suthee Rapisuwon, Benjamin Izar, Cory Batenchuk, Alexandre Avila, Shaolin Mei, Peter Sorger, Jerry M. Parks, Sarah J. Cooper, David Wagner, Jay C. Zeck, Aline J. Charabaty, Michael B. Atkins

**Affiliations:** 1 Department of Oncology, Lombardi Comprehensive Cancer Center, Georgetown University Medical Center, 3970 Reservoir Rd NW, New Research Building Suite E 501, Washington, DC 20057 USA; 2 Dana-Farber Cancer Institute, Boston, MA USA; 3 Bristol-Myers Squibb, Princeton, NJ USA; 4 Harvard Medical School-Harvard University, Boston, MA USA; 5 Medstar Georgetown University Hospital, Washington, DC USA; 6 Center for Molecular Biophysics, Biosciences Division, Oak Ridge National Laboratory, Oak Ridge, TN USA

## Abstract

Balancing the potential for durable remissions with autoimmune-like toxicities is a key clinical challenge in the use of immune checkpoint inhibitors (ICI). Certain toxicities are associated with an increased response rate; however, the molecular underpinnings of this association are poorly understood. Here, we report a patient with wide spread uveal melanoma who had an exceptional response to treatment with ipilimumab and nivolumab, but suffered severe immune-related sequelae, including central serous retinopathy with retinal detachment, tinnitus, and vitiligo resembling Vogt-Koyanagi-Harada disease, and refractory enteritis. TCR-sequencing of the primary tumor, a hepatic metastasis, duodenal biopsy and peripheral blood mononuclear cells, identified the identical T cell clone in all four tissues. This case provides preliminary evidence for cross-reactivity as a mechanism for the association between effect and toxicity of ICIs.

**Electronic supplementary material:**

The online version of this article (10.1186/s40425-019-0533-0) contains supplementary material, which is available to authorized users.

## Introduction

Uveal melanoma (UM) comprises < 3% of all melanomas with an incidence of 5–10 cases/million [1] and underlying biology that is distinct from cutaneous melanoma (CM). In the last decade, the interrogation of the genetic landscape [2] and advances in immuno-oncology [3] have led to a remarkable improved survival rate of 40–60% [4] in patients with metastatic CM. In contrast, patients with UM rarely (ORR 0–2.6%) [5, 6] respond to ICIs, including anti-CTLA-4 and anti-PD1 monotherapies, and show low response rate (15.8%) to the combination [7]. Intrinsic resistance to ICIs in UM may be related to various mechanisms, including a low somatic mutation rate [8] and paucity of tumor infiltrating lymphocytes [9]. In CM, ICI-related skin toxicities, such as rash and vitiligo, correlate with increased tumor response and prolonged survival [10]. The immunological underpinnings for this phenomenon in patients remain poorly understood. Delineating underlying mechanisms may help to identify approaches for dissociating treatment benefits and risks.

Here we report a patient with metastatic UM who experienced an exceptional response to dual blockade of PD-1 and CTLA-4. This response was accompanied by severe and unique immune-related adverse events (irAEs). Integrated analysis of several tissues, including primary tumor, a liver metastasis, inflamed duodenum and peripheral blood using whole-exome, transcriptome and T cell receptor (TCR) sequencing, and multiplexed immunofluorescence identified a dominant T cell clone. This report suggests that tumor-reactive T cell clones may play a role in mediating toxicity in healthy tissues.

## Case description

**Fig. 1:**
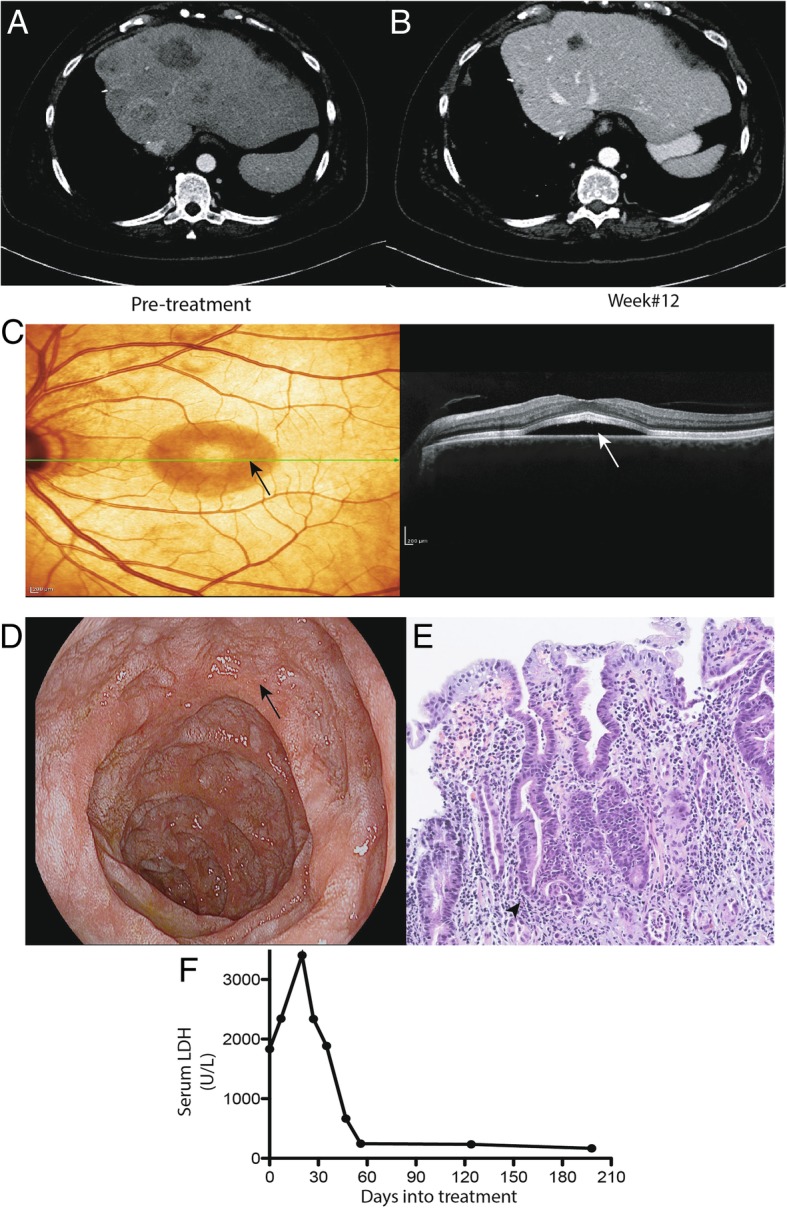
Clinical Characteristics. Panel **a** and **b** depict pre- and post-treatment computed tomography of the liver with complete resolution of liver metastases. Panel **c** depicts central serous retinopathy (arrow) on fundoscopic examination and Optical Coherence Tomography (OCT). Panel **d** and **e** shows endoscopic and pathologic findings of post-treatment duodenitis (arrow) with marked acute inflammatory cell infiltrate involving most of the glandular epithelium. The infiltrate is predominantly within deep crypt spaces (arrowhead). Panel **f** shows decline of serum LDH shortly after immunotherapy were initiated

A 60-year-old woman was diagnosed with 18 × 14 mm UM of the right eye and underwent enucleation in 2009. Pathology confirmed UM with monosomy 3 and 8q amplification. She developed a solitary hepatic metastasis in 2014 and underwent right hepatectomy. A multi-gene panel analysis of the tumor showed somatic BAP-1 and GNA11 mutations. She developed extensive metastases 9 months later with multiple hepatic, bone and lung lesions, and elevation of lactate dehydrogenase (LDH) > 1300 U/L. She received combination nivolumab and ipilimumab therapy. After two infusions, she developed central serous retinopathy of the left eye with retinal detachment, tinnitus and vitiligo resembling Vogt-Koyanagi-Harada (VKH) disease, an ocular autoimmune syndrome (Fig. 1c). CT scan at 12 weeks demonstrated significant reduction in hepatic metastases (Fig. 1a and b), and disappearance of lung and bone metastases. LDH level initially rose and then normalized (Fig. 1f). She continued on nivolumab monotherapy and experienced a near-complete response, but developed grade 3 duodenitis (Fig. 1d and e) requiring prolonged high-dose immunosuppressive therapy, including high-dose prednisone, followed by infliximab, and vedolizumab with eventual resolution. The clinical antitumor response persisted for over 1 year from treatment initiation and over 9 months from the last dose of immunotherapy. Unfortunately, she developed progressive brain and liver metastases after 1.5 year. Nivolumab monotherapy was resumed resulting in a mixed response and additional skin and eye toxicity, preventing further treatment. Due to overall declining health, the patient decided for supportive care and died 6 months after reinitiating original systemic therapy.

## Results

### Molecular and immunologic analyses

Tumor DNA from the liver lesion was sequenced at a depth of 60X and the PMBC sample was sequenced at 30X depth. Following data analysis and integration, a total of 111 somatic SNPs were identified (Additional file 1: Figure S1a). Twenty-one (19%) were predicted to be deleterious (moderate or high-impact) as they occurred within the coding region and did not result in a synonymous variant (Additional file 1: Table S1). Of these 21 mutations, only BAP1 and GNA11 were identified in the NCI Genomic Data Commons (GDC) UM dataset. The BAP1 gene, a prevalent mutation in UM [11] contained a stop-gain mutation. GNA11, a G-protein-coupled receptor, contained the Q209L mutation present in 33 of 80 UM annotated by GDC. The remaining commonly mutated genes in UM, e.g., EIF1AX, SF3B1, and GNAQ, were wild-type. None of the 21 deleterious mutations have been associated with vitiligo or VKH. The rate of SNVs resulting in potential neo-epitopes (21 SNVs) was similar to the rate described in the GDC UM dataset (median: 15) (Additional file 1: Figure S2) as well as for subjects with BAP1 mutation (median: 14) or stage III/IV disease (median: 13). In comparison, the mutation rates in CM from patients with stage III/IV disease [12] are ~ 25 fold greater.

To better understand the tumor microenvironment, we compared the RNA signature from the liver metastasis to the RNA signature for the 80 primary UM in GDC. Based on this comparison, the liver metastasis was predicted to have brisk immune cell infiltration (Additional file 1: Figure S3). The gene signature of the patient’s sample was the top ranked sample defined by the median reading for macrophage genes (CD163, IL4R & CD68), the 5th highest for genes associated with cytotoxic T lymphocytes (CD3D, CD3E, CD3EAP, CD3G, CD8A & CD8B) and the 22nd highest for genes expressed by NK cells (SLAMF7, KLRK1 & GZMB). Similarly, the sample was the 10th highest with respect to average expression of an IFNγ signature (CXCL10, CXCL9, STAT1, IDO1, IFNγ) previously associated with response to immunotherapy [13]. Both vitiligo associated genes (PMEL, TYRP1, DCT & MLANA) and PD-L1 had mRNA levels near the median value observed across the dataset. Of the 21 mutated loci, 10 were qualified as associated with the upper quartile of gene expression relative to the GDC dataset (TRIP11, ZNF326, PKD1L2, CHRM2, SF3B1, CSE1L, EIF1AX, CHN2, SRRM2 & CATSPERG).

**Fig. 2:**
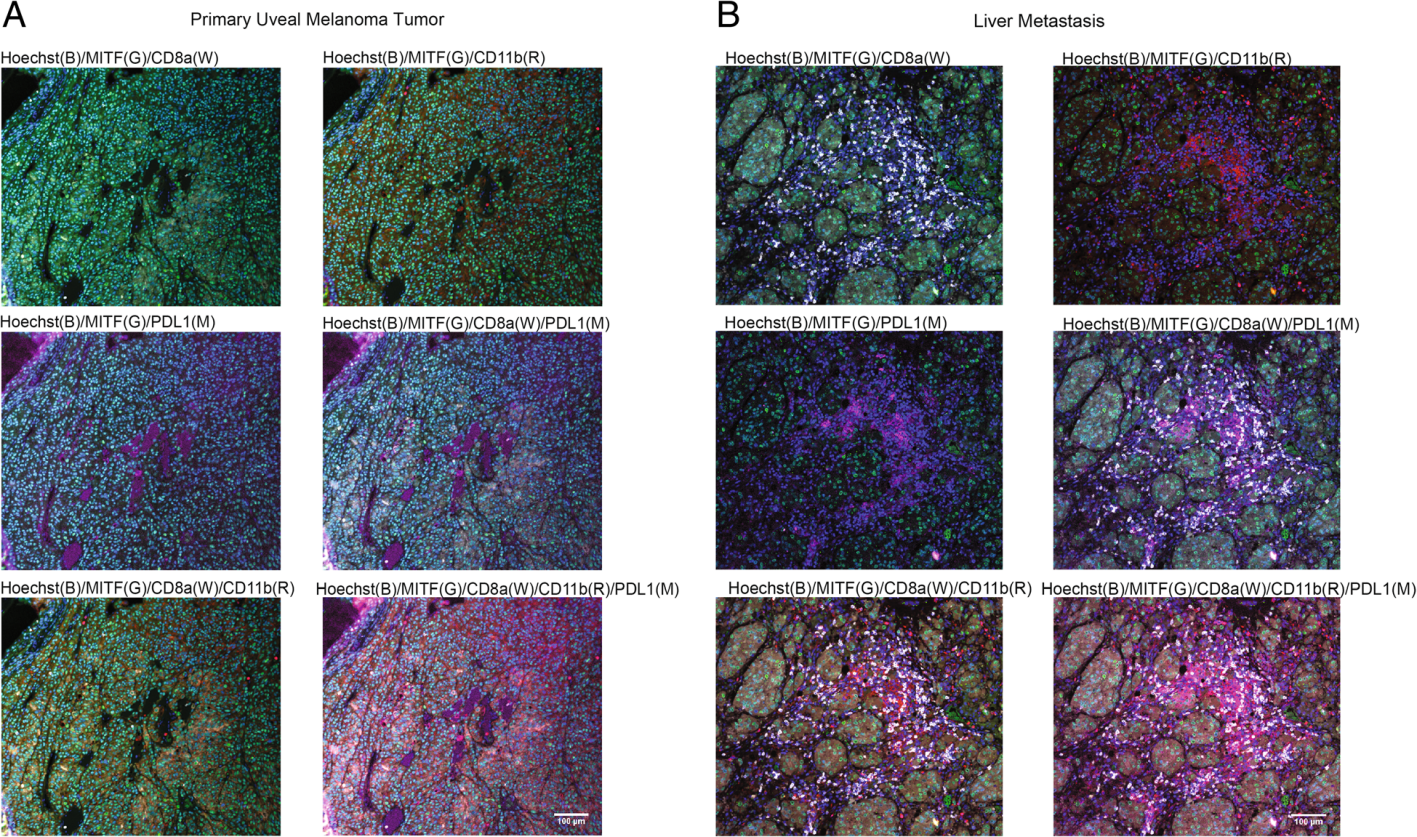
**a** and **b** Multiplexed immunofluorescence of hepatic metastasis. Staining of nuclei (Hoechst) MITF, PD-L1, CD8a and CD11b is shown at 20X magnification. Letters indicate respective color representation in the images, including blue (B), green (G), white (W), red (R) and magenta (M). Different staining combinations are shown and indicated at the top of each image. MITF expression identifies cancer cells. From top left to bottom right, the images show significant infiltration with CD8a and CD11b expressing immune cells and elimination of tumor cells in the areas of immune infiltrates. PD-L1 expression is almost exclusively limited to myeloid infiltrating immune cells (CD11 + PD-L1+). The primary tumor was imaged using the same strategy (see Additional file 1: Supplemental method)

Based on RNA expression data, we subsequently investigated the immunobiology landscape of the primary eye tumor and liver tumor tissues using CycIF [14]. The primary tumor contained very few PD-L1+ stromal cells (magenta) and CD8 + T cells (white), with low levels of CD11b + macrophages (red) (Fig. 2). The liver tumor, despite low level of PD-L1+ stromal cells and widely expressed CD11b + macrophages, exhibited an intense immunofluorescent signal of CD8+ T cells (Fig. 2b). This finding suggests naturally occurring tumor-specific T cell recognition and expansion of TILs in the liver metastasis.

**Fig. 3:**
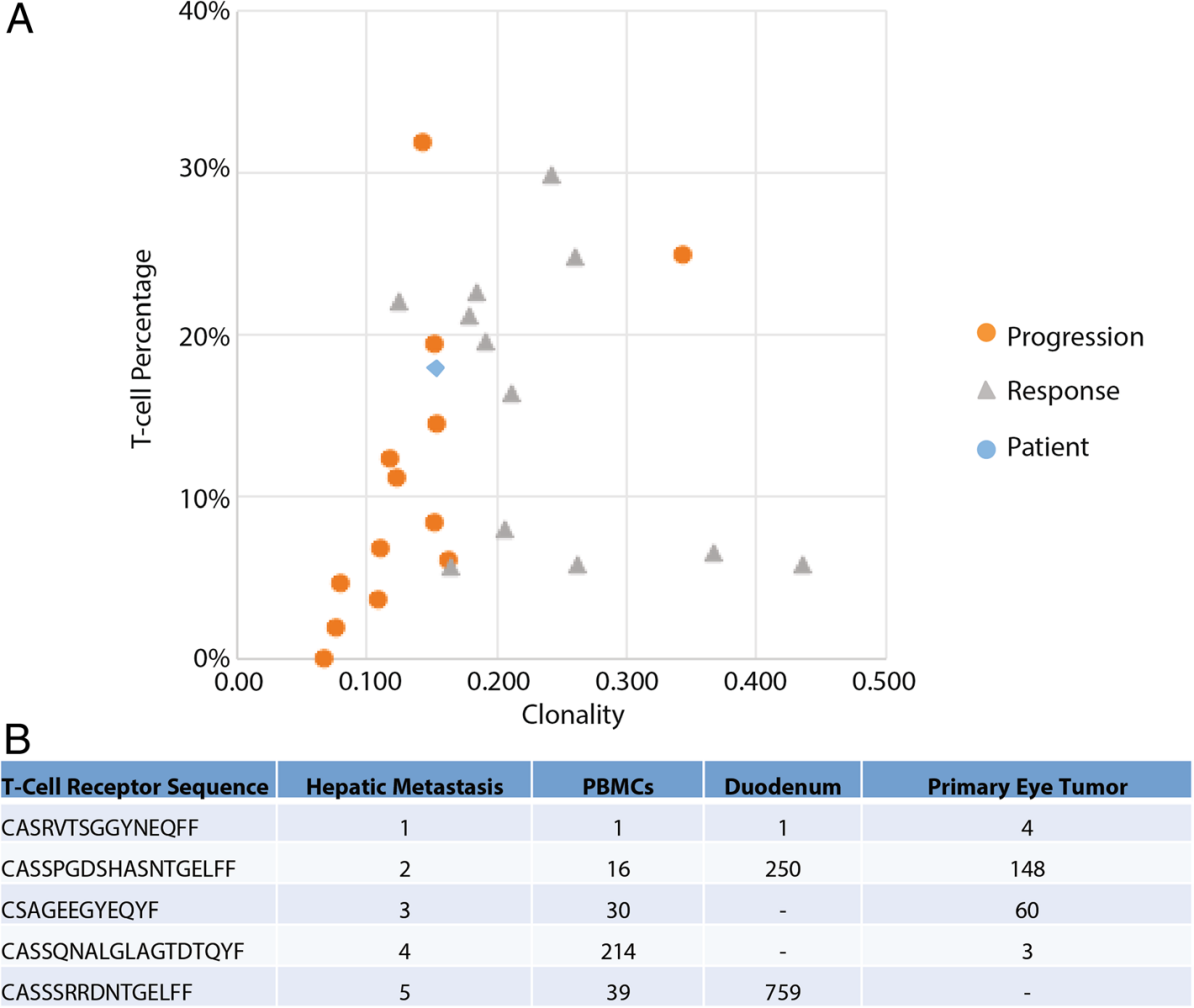
Expansion of clonal T-cells from Primary Uveal Melanoma. Panel **a** depicts TCR clonality metrics in the TME relative to published result regarding the association of these metrics with recently reported responses to anti-PD1 therapy in cutaneous melanoma [15]. Panel **b** depicts amino acid sequence of corresponding uniquely rearranged variable TCR β-chain regions in primary eye tumor, hepatic metastasis, PBMCs, and inflamed duodenum. TCR sequence, “CASRVTSGGYNEQFF” was expanded from the primary eye tumor (4^th^most prevalent) to become the most abundant clone in hepatic metastasis, and peripheral blood that persisted more than 6 months after treatment initiation. The same clone is seen as the top infiltrating T-cell clone in duodenal crypt inflammation

TCR sequencing confirmed immune involvement in the metastatic tissue prior to therapeutic intervention. There were an estimated 39,794 T cells of 218,816 nucleated cells (18%). The clonality metric was 0.153, indicating a clonal T-cell population against a specific tumor antigen. When compared to the CM dataset generated by Tumeh et al. [15], both T-cell abundance and T-cell clonality indicated that the patient was likely to respond to therapy given the above median values for both metrics (Fig. 3a). To better understand the TCR clones involved, the primary tumor sample, and a duodenal sample and PBMC samples collected during treatment were submitted for TCR sequencing (Adaptive). Of the top 10 sequences detected in the primary tumor sample, many were among the top TCR sequences detected in the additional samples (Fig. 3b). In particular, the “CASRVTSGGYNEQFF” amino acid sequence derived from TCRBV19–01 represented the most prevalent clone in the metastatic tumor, PBMC and duodenal samples. In the primary lesion obtained several years prior, it was the 4th highest sequence detected. We did not characterize the specificity of this TCR sequence further.

To determine the potential for each candidate neoantigen to be presented by the subject’s class-I MHC and bind to the identified clonal T-cells, we performed in-silico binding affinity predictions between the HLA allele (HLA-A*24:02) and all possible 8- to 11-mer peptides that included mutation-related residues. Using NetMHC 4.0 [16] and NetMHCpan 4.0 [17], none of the peptide candidates was predicted to be a suitable binder (IC_50_ ≤ 500 nM) to the patient’s HLA allele.

### Pharmacovigilance

A search of the BMS safety database revealed 8 cases with the reported term of VKH. Two cases involved ipilimumab monotherapy, one involved ipilimumab in combination with nivolumab, three involved nivolumab monotherapy, one was treated with nivolumab followed by ipilimumab, and another nivolumab followed by vemurafenib. Five of these cases have been reported in the literature [18–21]. Five were from Japan, two from the USA, and one from Germany. Two of the nivolumab monotherapy cases from Japan were patients with NSCLC. The other six cases had melanoma. No cases had UM.

## Discussion

This exceptional response of the patient’s UM to combined ICI illustrates the potential critical role of clonal expansion of TILs in achieving clinical benefit from immunotherapy. That the same CD8+ TIL clones in the primary tumor expanded in the metastases suggests that these TILs were directed against the same antigen on the tumor. Detection of this clone in PBMC and within the duodenum, a site of irAE, strongly suggests that the antitumor effects and toxicity were mediated by the same T cell clone. The involvement of barrier tissues (skin, gastrointestinal mucosa) by irAEs beyond drug exposure supports the possibility that adverse effects may be mediated by tissue-resident memory T cells (TRM) [22] Further, the presence of a specific TCR clone in the primary eye tumor, liver metastasis and site of enteritis and the occurrence of VKH and vitiligo suggest that the clone was directed against a shared antigen/epitope rather than a tumor neoantigen. In the absence of neoantigens that strongly bind class-I MHC, the irAEs may stem from enhanced TCR interactions with self-antigens arising from ICI therapy.

When anti-tumor reactivity is directed against a shared antigen the therapeutic index may be narrow, with toxicity occurring nearly simultaneously with antitumor response, as in this case. This observation has been recently reported in another case of metastatic UM that achieved a durable major response to the combination ICI in association with autoimmune hepatitis, uveitis and exocrine pancreatic insufficiency [23].

While activation of quiescent immune-reactive T cells resident in non-tumor tissue is purported to be the mechanism of autoimmune thyroiditis, colitis, and other typical toxicities of ICI, the irAEs in both cases of advanced UM suggest that there was cross-reactivity of T cell immunity against an antigen that was shared between the UM and the contralateral uvea, the skin and non-tumor organs. That the duodenitis was unresponsive to steroids and infliximab, but did respond promptly to vedolizumab [24], a monoclonal antibody to integrin α4β7 (LPAM-1), which blocks T cell infiltration into the GI tract, suggests that her GI toxicity derived from circulating T cells that trafficked into the GI tract following expansion in a distant site, such as the tumor. A similar pathophysiology may occur in the cases of extreme cardiotoxicity, where expanded T cell clones that were present in the tumor were also identified in the inflamed heart musculature [25].

Thus, the value of a high mutational load may be to induce immunoreactivity against multiple neoantigens and simultaneously overwhelm any shared antigen response, thereby eliminating the tumor before significant shared antigen expansion and related organ toxicity develops. Such biology might explain the enhanced durable antitumor benefit observed in patients with irAEs, despite the use of often-prolonged immunosuppressive therapy.

Ultimately, it may be possible, albeit difficult, to identify at baseline patients with expanded immunity against antigens shared by the tumor and vital host organs, so that one could take measures to minimize the organ toxicity. Alternatively one could use pre-treatment vaccination against neo-epitopes in order the steer the immune response towards tumor restricted rather than shared antigens.

This report emphasizes that while the response to ICI is significantly lower in patients with UM compared to those with CM, a major response can be achieved. Additional work is required to select the appropriate patients with UM who are destined to benefit from ICI and to determine a means of dissociating anti-tumor responses against shared antigens from toxicity to organs expressing the same antigens.

## Additional file

Additional file 1: Supplemental Appendix. (DOCX 7 kb)
