# Supplementary material for: Exceptional response and multisystem autoimmune-like toxicities associated with the same T cell clone in a patient with uveal melanoma treated with immune checkpoint inhibitors
Source: J Immunother Cancer. 2019 Mar 4;7:61. doi: 10.1186/s40425-019-0533-0 (PMC6399858; doi:10.1186/s40425-019-0533-0)
Supplement: Supplementary file 1 — Supplemental Appendix. (DOCX 7 kb) [file 40425_2019_533_MOESM1_ESM.docx]

Table of Contents

Supplemental Appendix 2

Supplemental Methods: 2

Multiplexed Immunofluorescence 2

Sample Preparation: 2

Whole exome sequencing: 3

RNA sequencing 3

T-cell receptor variable beta chain sequencing 5

Supplemental Figure Legends: 5

Supplemental Data Figure 1 5

Supplemental Data Figure 2: 5

Supplemental Data Figure 3: 6

Supplemental Data Table 1 6

Supplemental Data Table 2 6

Supplemental Figures and Tables 6

Supplemental Data Figure 1 6

Supplemental Data Figure 2 7

Supplemental Data Figure 3 7

Supplemental Data Table 1 8

Supplemental Data Table 2: Immunofluorescence Antibody List and Dilution 9

Reference: 9

# Additional file 1

# Supplemental Appendix

## Supplemental Methods:

### Multiplexed Immunofluorescence

Tumor tissue and formalin-fixed, paraffin-embedded (FFPE) were collected from this patient under IRB-approved protocols 2014-1013 at Georgetown University. We used, tissue CyCIF for multiplexed immunofluorescence (IF), we used a recently developed technology, tissue CyCIF^1^. Routine FFPE specimens (5 µm thick cuts mounted on a glass slide) are initially dewaxed, rehydrated and stained by indirect IF and Hoechst 33342, followed by image acquisition on a RareCyte CyteFinder instrument (RareCyte Inc. Seattle WA). Fluorophores are then inactivated with 4.5% H_2_O_2_ and 24 mM NaOH made in PBS for 1 hour at RT in the presence of white light. Subsequently, the same tissue slide is stained by direct IF and this cycle is repeated until all images are acquired. Antibodies were used at indicated dilutions (supplemental data table 2).

### Sample Preparation:

The tumor sample from Formalin-Fixed, Paraffin-Embedded (FFPE) slides and non-tumor sample fresh-frozen peripheral blood were shipped directly from the study site to Macrogen (Rockville, MD) for processing. Non-tumor DNA was extracted from blood samples obtained during study clinic visits, and tumor RNA/DNA was extracted from FFPE preserved tissue from a liver metastatic lesion containing a mix of cancerous and non-cancerous material. Exclusively for TCR sequencing, DNA was equally extracted from the primary lesion and duodenal tissue.

For Whole Exome Sequencing, DNA quality was assessed in two steps. First the DNA was purified and separated by size using gel electrophoresis. Images of the gel were visually inspected for signs of fragmentation. Samples passing visual inspection had their fragmentation quantified using a DIN score, which is a quality scale ranging from 1-10 calculated from several features of the electrophoretic trace [ ] and was associated with a DIN score of 7 or higher. DNA Library preparation was performed using the Agilent SureSelect V4-post capture kit according to the Agilent SureSelect Target Enrichment Kit preparation guide.

For RNA-Sequencing, DNA contamination was removed using DNase. Library preparation was done using the TruSEq RNA Access Library Prep Kit. The purified RNA was randomly fragmented and reverse transcribed into cDNA, then ligated to adapters. After PCR amplification, fragments with insert sizes 200-400bp were selected for paired-end sequencing.

### Whole exome sequencing:

For Whole Exome Sequencing, DNA quality was assessed in two steps. First the DNA was purified and separated by size using gel electrophoresis. Images of the gel were visually inspected for signs of fragmentation. Samples passing visual inspection had their fragmentation quantified using a DIN score, which is a quality scale ranging from 1-10 calculated from several features of the electrophoretic trace [ ] and was associated with a DIN score of 7 or higher. DNA Library preparation was performed using the Agilent SureSelect V4-post capture kit according to the Agilent SureSelect Target Enrichment Kit preparation guide.

DNA sequencing was performed on the HiSeq4000 sequencer.

#### Data handling:

Following sequencing, alignment to the HG19 reference genome was performed using BWA (Burrows-Wheeler Alignment Tool) package ^2^, and consists of three algorithms : BWA-backtrack, BWA-SW and BWA-MEM. Picard was used for to remove duplicate fragments. The Genome Analysis Toolkit (GATK) was used for indel realignment and variant calling/filtering ^3^.  HaplotypeCaller was used to identify SNPs and indels simultaneously via local re-assembly of haplotypes. Variant annotations inferred  using SnpEff - an open-source tool for variant annotation and effect prediction ^4^. Control-FREE Copy Number and Genotype Caller (Control-FREEC) was used for CNV analysis of the tumor and non-tumor samples, then ExomeCNV used for the tumor-non-tumor comparison. MuTect was used to pre-processes the aligned reads to remove reads with too many mismatches or low quality scores, then uses two Bayesian classifiers to predict sites likely to carry somatic mutations with high confidence ^5^.

### RNA sequencing

For RNA-Sequencing, DNA contamination was removed using DNase. Library preparation was done using the TruSEq RNA Access Library Prep Kit. The purified RNA was randomly fragmented and reverse transcribed into cDNA, then ligated to adapters. After PCR amplification, fragments with insert sizes 200-400bp were selected for paired-end sequencing.

RNA sequencing was performed on the HiSeq4000 sequencer.

#### Data handling:

Following RNA-Sequencing, quality control was performed using FastQC v0.10.0. Low quality reads, adaptor sequences, PCR duplicates, and contaminant DNA sequences were removed using Trimmomatic v0.32. The trimmed reads were mapped to reference genome (USCG hg19) using Bowtie2 v.2.2.3 ^6^ and TopHat v.2.0.13 ^7^ a splice-aware aligner. The transcripts were assembled using Cufflinks (v2.2.1) ^8^ with aligned reads that contained paired-end information. Fusion genes were predicted from RNAseq results using defuse v0.6.2, which clusters discordant paired-end alignments (spanning reads and split reads) to predict the correlation between a fragments’s length distribution and split reads. A heuristic filter is applied to analyze the correlation and predict the existence of fusion genes. Gene expression profiles were compared to the Uveal Melanoma Dataset deposited in NCI Genomic Data Commons (GDC) which was analyzed under default parameters and aligned to the reference genome (GRCh38). All genes mapping to multiple ensemble identifiers in the GDC dataset were removed from further analysis. Identification of liver specific genes was performed using the read counts FANTOM5 Table ExtractionTool (http://fantom.gsc.riken.jp/5/tet/#!/search/hg19.cage_peak_counts_ann_decoded.osc.txt.gz?c=5&c=663&c=810) to obtain liver (liver, adult, pool1; CNhs10624.10018-101C9) and retina (retina, adult, pool1; CNhs10636.10030-101E3) datasets ^9^. The sum of read counts mapping to a specific ensembl identifier was used to define gene expression. All genes absent within the dataset or mapping to multiple ensemble identifiers in the Fantom 5 dataset were removed from further analysis.  Tissue specific genesets were defined as 5 fold threshold between the two datasets. Following removal of liver and retina specific genes, the FPKM values of the N-of-1 sample was integrated with the GDC Uveal Melanoma Datasets and quantile normalized.

The results for the cutaneous melanoma and UVM datasets deposited in GDC were in whole based upon data generated by the TCGA Research Network: <http://cancergenome.nih.gov/>. RNA sequencing experiments were downloaded from GDC while high-impact mutations were abstracted from: http://www.cbioportal.org. Inference of HLA-type was performed using BWA-KIT (v7.12).

### T-cell receptor variable beta chain sequencing

Immunosequencing of the CDR3 regions of human TCRβ chains was performed using the ImmunoSEQTM Assay (Adaptive Biotechnologies, Seattle, WA). Extracted genomic DNA was amplified in a bias-controlled multiplex PCR, followed by high-throughput sequencing. Sequences were collapsed and filtered in order to identify and quantitate the absolute abundance of each unique TCRβ CDR3 region for further analysis as previously described ^10-12^

#### Statistical Analyses of TCR-β sequencing results

Clonality was defined as 1- Peilou’s eveness ^13^ and was calculated on productive rearrangements by: where pi is the proportional abundance of rearrangement i and N is the total number of rearrangements. Clonality values range from 0 to 1 and describe the shape of the frequency distribution: clonality values approaching 0 indicate a very even distribution of frequencies, whereas values approaching 1 indicate an increasingly asymmetric distribution in which a few clones are present at high frequencies. Statistical analysis was performed in R version 3.2.

For TCR-β sequencing from FFPE tissues, the fraction of T cells in FFPE tissue samples was calculated by normalizing TCR-β template counts to the total amount of DNA usable for TCR sequencing, where the amount of usable DNA was determined by PCR-amplification and sequencing of several housekeeping genes that are expected to be present in all nucleated cells.

## Figure Legends:

### Figure S1. SNV Characterization

Classification of SNVs detected in the patient. * Denotes high- and mid-impact mutations affecting protein structure & function. Very few mutations were detected in this patient, where 21 loss-of-function mutations were observed and 20 would have resulted in the formation of a potential tumor antigen.

### Figure S2: Mutation Count Comparison

Deleterious mutation count detected in the patient (red line) relative to all primary uveal melanoma (n=80), primary uveal melanoma from stage harboring a BAP1 mutation (n=26), primary uveal melanoma from stage from stage III/IV patients (n=43), all cutaneous melanoma (n=363) & cutaneous melanoma from stage III/IV patients (n=163).

### Figure S3: Gene Expression Heatmap

Heatmap of quantile normalized gene expression of known biomarkers for immunotherapy and vitiligo. Heatmap color intensity represents the fold change of the expression value over the median value for the population. X-axis represents the case study samples (CS) relative to all 80 measurements of these genes in uveal melanoma (1-80).

### Table S1 Gene Variants and Mutation Frequency

Table summarizing gene variants detected in present case study by WES relative to all uveal melanoma patients deposited in TCGA (n=80) and in cutaneous melanoma according to TCGA (n=269) according to Illumina cohort analyzer. The mutational profile is consistent with literature, of the 5 high frequency mutations in uveal melanoma, 2/5 were detected.

### Table S2 Immunofluorescence Antibody List and Dilution

Table summarizing the immunofluorescent antibodies used in the case study including manufacturer, product ID and antibody dilution.

## Figures and Tables

### Figure S1

### Figure S2

### Figure S3

### Table S1

###

### Table S2: Immunofluorescence Antibody List and Dilution

| **Antibodies** | **Manufacturer** | **Product ID** | **Dilution** |
| --- | --- | --- | --- |
| F(ab’)2-goat anti-rabbit IgG-488 | ThermoFisher | A-11070 | 1:2000 |
| donkey anti-goat IgG-555 | ThermoFisher | A-21432 | 1:2000 |
| F(ab’)2-goat anti-mouse IgG-647 | ThermoFisher | A-21237 | 1:2000 |
| S100α-488 | Abcam | ab207367 | 1:2000 |
| CD3-555 | Abcam | ab208514 | 1:300 |
| HLA-A-647 | Abcam | ab199837 | 1:1000 |
| MITF-488 | Abcam | ab201675 | 1:300 |
| CD45-PE | R&D Systems | FAB1430P | 1:150 |
| PD1-647 | Abcam | ab201825 | 1:200 |
| CD4-488 | R&D Systems | FAB8165G | 1:150 |
| FOXP3-e570 | ThermoFisher | 41-4777-82 | 1:150 |
| PDL1-647 | CST | 15005 | 1:200 |
| CD56-488 | Abcam | ab200333 | 1:150 |
| CD11b-555 | Abcam | ab206616 | 1:400 |
| CD8a-e660 | ThermoFisher | 53-0008-82 | 1:150 |

# References:

1. A simple open-source method for highly multiplexed imaging of single cells in tissues and tumours. Cold Spring Harbor Laboratory, 2017. 2017, at <https://www.biorxiv.org/content/early/2017/06/19/151738>.)

2. Li H, Durbin R. Fast and accurate short read alignment with Burrows-Wheeler transform. Bioinformatics 2009;25:1754-60.

3. McKenna A, Hanna M, Banks E, et al. The Genome Analysis Toolkit: a MapReduce framework for analyzing next-generation DNA sequencing data. Genome Res 2010;20:1297-303.

4. Variant annotations in VCF format. 2018. at <http://snpeff.sourceforge.net/VCFannotationformat_v1.0.pdf>.)

5. Cibulskis K, Lawrence MS, Carter SL, et al. Sensitive detection of somatic point mutations in impure and heterogeneous cancer samples. Nat Biotechnol 2013;31:213-9.

6. Langmead B, Salzberg SL. Fast gapped-read alignment with Bowtie 2. Nat Methods 2012;9:357-9.

7. Kim D, Pertea G, Trapnell C, Pimentel H, Kelley R, Salzberg SL. TopHat2: accurate alignment of transcriptomes in the presence of insertions, deletions and gene fusions. Genome Biol 2013;14:R36.

8. Trapnell C, Williams BA, Pertea G, et al. Transcript assembly and quantification by RNA-Seq reveals unannotated transcripts and isoform switching during cell differentiation. Nat Biotechnol 2010;28:511-5.

9. Hon CC, Ramilowski JA, Harshbarger J, et al. An atlas of human long non-coding RNAs with accurate 5' ends. Nature 2017;543:199-204.

10. Robins H, Desmarais C, Matthis J, et al. Ultra-sensitive detection of rare T cell clones. J Immunol Methods 2012;375:14-9.

11. Carlson CS, Emerson RO, Sherwood AM, et al. Using synthetic templates to design an unbiased multiplex PCR assay. Nat Commun 2013;4:2680.

12. Robins HS, Campregher PV, Srivastava SK, et al. Comprehensive assessment of T-cell receptor beta-chain diversity in alphabeta T cells. Blood 2009;114:4099-107.

13. Kirsch I, Vignali M, Robins H. T-cell receptor profiling in cancer. Mol Oncol 2015;9:2063-70.
